# Renin-angiotensin inhibitors reduce thrombotic complications in Essential Thrombocythemia and Polycythemia Vera patients with arterial hypertension

**DOI:** 10.1007/s00277-023-05417-w

**Published:** 2023-08-21

**Authors:** Olga Mulas, Brunella Mola, Alessandro Costa, Francesca Pittau, Daniela Mantovani, Samuele Dessì, Antonella Fronteddu, Giorgio La Nasa, Giovanni Caocci

**Affiliations:** 1 Department of Medical Sciences and Public Health, University of Cagliari, Cagliari, Italy; 2 Hematology Unit, Businco Hospital, ARNAS Brotzu Cagliari, Cagliari, Italy

**Keywords:** MPNs, Hypertension, Thrombotic events, Renin-Angiotensin System

## Abstract

Essential Thrombocythemia (ET) and Polycythemia Vera (PV) are chronic myeloproliferative neoplasms (MPNs) characterized by thrombotic and hemorrhagic complications, leading to a high risk of disability and mortality. Although arterial hypertension was found to be the most significant modifiable cardiovascular (CV) risk factor in the general population, little is known about its role in MPNs as well as a possible role of renin-angiotensin system inhibitors (RASi) in comparison with other anti-hypertensive treatments. We investigated a large cohort of 404 MPN adult patients, 133 diagnosed with PV and 271 with ET. Over half of the patients (53.7%) reported hypertension at MPN diagnosis. The 15-year cumulative incidence of thrombotic-adverse events (TAEs) was significantly higher in patients with hypertension (66.8 ± 10.3% vs 38.5 ± 8.4%; HR = 1.83; 95%CI 1.08–3.1). Multivariate analysis showed that PV diagnosis and hypertension were independently associated with a higher risk of developing TAEs (HR = 3.5; 95%CI 1.928–6.451, *p* < 0.001 and HR = 1.8; 95%CI 0.983–3.550, *p* = 0.05, respectively). In multivariate analysis, the diagnosis of PV confirmed a significant predictive role in developing TAEs (HR = 4.4; 95%CI 1.92–10.09, *p* < 0.01), also considering only MPN patients with hypertension. In addition, we found that the use of RASi showed a protective effect from TAEs both in the whole cohort of MPN with hypertension (HR = 0.46; 95%CI 0.21–0.98, *p* = 0.04) and in the subgroup of thrombotic high-risk score patients (HR = 0.49; 95%CI 0.24–1.01, *p* = 0.04). In particular, patients with ET and a high risk of thrombosis seem to benefit most from RASi treatment (HR = 0.27; 95%CI 0.07–1.01, *p* = 0.03). Hypertension in MPN patients represents a significant risk factor for TAEs and should be adequately treated.

## Introduction

Essential Thrombocythemia (ET) and Polycythemia Vera (PV) are chronic Philadelphia-negative myeloproliferative neoplasms (MPNs) characterized by elevated blood cell proliferation and potential evolution into myelofibrosis (post-ET and post-PV MF) and acute myeloid leukemia (AML) [1]. It is common for individuals with MPN to experience microvascular symptoms and to present a higher risk of thrombotic adverse events (TAEs). In particular, patients with ET are more susceptible to arterial TAEs, while PV patients may experience venous TAEs more frequently. When assessing the risk of thrombotic complications in patients with PV and ET, age and history of thrombosis have been traditionally used as predisposing factors [2–4]. Patients under 60 years without a history of thrombosis are considered at low risk, while those with age ≥ 60 years or that suffered a previous thrombosis are classified as high-risk [5, 6].

Patients in the low-risk category are suggested to start an antiplatelet treatment using low doses of aspirin or similar medications; additionally, individuals with PV and hematocrit (HCT) levels above 45% should undergo periodical phlebotomy to keep HCT < 45%. Patients belonging to the higher-risk category are recommended to start a cytoreductive treatment to maintain platelet (PLT) level < 400 × 10^9^/L and HCT level < 45% [7], besides antiplatelet therapy.

In most cases, hydroxycarbamide is the preferred option for cytoreductive treatment. However, in certain circumstances, it may be necessary to use alternative drugs like interferon, particularly for patients who are of childbearing age [8]. For those patients intolerant or resistant to hydroxycarbamide, other medications like anagrelide or ruxolitinib may be reserved [9, 10].

The European Leukemia Network (ELN) recommendations for 2021 have highlighted the role of leukocytosis in PV as an extra risk factor for thrombosis. They have also proposed that a cytoreductive treatment should be initiated in the low-risk category group when leukocytosis is present [11]. In addition, mutational status was suggested to play an essential role in defining the risk category. Indeed, JAK2V617F mutation in ET was associated with the high-risk category in the IPSET-revised score system [12]. Modifiable factors such as hypertension, diabetes, dyslipidemia, and body mass index are considered classical cardiovascular (CV) risk factors that lead to an increased risk of cardiovascular adverse events (CV-AEs) [13–17]. It is widely acknowledged that hypertension and CV-AEs are closely linked in the general population. However, limited evidence supports this association in MPNs and TAEs [18]. Recent reports have addressed the role of hypertension in PV patients, but, to our best knowledge, no data has been reported in ET [19, 20].

Furthermore, there are no data on the role of specific categories of antihypertensive drugs and the prevention of TAEs in MPNs patients. A recent report has drawn attention to the potential benefits of using renin-angiotensin inhibitors (RASi) in preventing arterial occlusive events in patients with chronic myeloid leukemia, compared with other antihypertensive drugs [21]. Further research may be required to determine the effectiveness of these drugs in preventing TAEs in MPN patients. Due to limited available data in this area, our study aimed to evaluate the relationship between hypertension at MPN diagnosis and the incidence of TAEs in a large group of patients. Additionally, we explored the potential advantages of using RASi to reduce the incidence of TAEs in PV and ET patients.

## Patients and methods

We retrospectively evaluated patients diagnosed with PV or ET according to WHO 2016 classification [1], attending from November 2000 through August 2021, the Hematology Unit of the Businco Hospital in Cagliari, Italy.

PV patients were stratified according to the risk of developing thrombosis in low-risk (age < 60 years and no history of thrombosis) and high-risk (age ≥ 60 years or with a history of thrombosis). Additionally, ET patients were stratified by the IPSET score, considering CV risk factors, age > 60 years, thrombosis history, and presence of JAK2 V617F mutation. The following clinical data at MPN diagnosis were collected: constitutional symptom, performance status, hemoglobin (Hb), HCT, PLT, neutrophils, white blood cell (WBC) counts, serum lactate dehydrogenase (LDH), erythropoietin level, bone marrow biopsy, presence of somatic driver gene mutations (JAK2V617F, Calreticulin, MPL), splenomegaly, karyotype when available, phlebotomies or other drug treatment. The past clinical history and anamnestic report of arterial or venous thrombosis or other cardiovascular adverse events (CV-AEs) were collected at baseline. Arterial CV-AEs included arterial hypertension, angina, stroke, myocardial infarction, heart arrhythmia, heart failure, aortic aneurysms, cardiomyopathy, ischemic cerebrovascular events, thromboembolic disease, valvular heart, or peripheral artery disease. Venous CV-AEs were mainly represented by venous thrombosis.

Other CV risk factors were also considered, such as diabetes, dyslipidemia, increased body mass index (BMI) > 24.5 kg/m2, or severe renal insufficiency. Primary or secondary antithrombotic prophylaxis was reported. Anti-hypertensive treatment in patients with hypertension was stratified according to drug categories: RASi included angiotensin-converting enzyme inhibitors (ACEi) and angiotensin receptors blockers (ARBs); other medications were represented by calcium antagonists or other drugs such as thiazide diuretics, beta-blockers, and doxazosin.

Percentages derived from univariate analysis were compared using the *X*^*2*^ test. Probabilities of cumulative incidence of CV-AEs were estimated by the Kaplan–Meier method; the log-rank test was used to compare two or more groups of stratified patients. Multivariate analysis was performed with a Cox proportional hazards regression model, considering TAEs as the dependent variable and the following independent variables: age, diagnosis of PV or TE, sex, dyslipidemia, and hypertension at MPN diagnosis. Multivariate analysis in the group of patients with hypertension considered as independent variables the thrombotic risk score, the PV diagnosis, the age, sex, use of statin ad diagnosis, and treatment with RAS inhibitors vs other anti-hypertensive drugs. The study was conducted by the Declaration of Helsinki and was approved by the local ethical committee. Patients signed informed consent under a protocol approved by Cagliari Ethics Board (PROT. PG/2021/8545).

## Results

Patient characteristics are shown in Table 1. A total of 404 MPN patients were enrolled in the study. Among these, 217 (53.7%) reported hypertension before MPN diagnosis. One hundred and thirty-three (32.9%) were diagnosed with PV, and 271 (67.1%) with ET. The median age at diagnosis was 63 years (17–98), and 50% of patients were male. The median follow-up was 5.5 years (range 0–24). Overall, 361 (89.3%) patients presented a gene mutational status. Jak2V617F mutation was detected in 317 (78.5%) patients and was significantly higher in the group of patients with hypertension (45 vs 33.1%, *p* < 0.01). Calreticulin and MPL mutations were present in 36 (13.2%) and 6 (2.2%) patients. There was a significant difference in the Jak2V617F mutation status within the group of ET patients with hypertension (27% vs 21.2%, *p* = 0.01). At MPN diagnosis, 268 (66.3%) patients reported CV-AEs events; they were more frequent in patients with hypertension (53.5% vs 12.8%, *p* < 0.01). The following TAEs were detected: 31 ischemic heart disease, 14 peripheral arterial occlusive disease, 28 cerebrovascular events, and 19 deep vein thrombosis.

**Table 1:** Characteristics of patients with Polycythemia Vera and Essential Thrombocythemia, according to the hypertensive status

	Total	With hypertension	Without hypertension	*p* value
MPNs patients, *N* (%)	404 (100)	217 (53.7)	187 (46.3)	
Polycythemia Vera, *N* (%)	133 (32.9)	78 (19.3)	55 (13.6)	ns
Essential Thrombocythemia, *N* (%)	271 (67.1)	139 (34.4)	132 (32.7)	ns
Sex male, *N* (%)	202 (50)	102 (25.3)	100 (24.7)	ns
Age at diagnosis, median years (range)	63 (17–98)	68.5 (28–94)	56.6 (23–95)	< 0.01
Follow-up, median years (range)	5.5 (0–24)	4.8 (0–22)	5.7 (0–24)	< 0.01
Leukocyte × 10^3^/μL, median value (range)	10.5 (1.0–96.1)	10.9 (1.09–19.2)	9.9 (1.0–96.1)	ns
Hemoglobin g/dL, median value (range)	15.0 (7.0–15.0)	15.2 (10.4–21.0)	14.7 (7.0–19.0)	ns
Hematocrit,	48 (29.0–77.0)	47.6 (33.1–69)	48.1 (29.0–69)	ns
Platelet × 10^3^/μL, median value (range)	696 (87–2320)	720 (139–1770)	702 (100–2320)	ns
Positive mutational status, *N* (%)	361 (89.3)	197 (48.7)	161 (39.8)	ns
*Jak2V617F*	317 (78.5)	182 (45)	134 (33.1)	< 0.01
*Calreticulin*	36 (8.9)	12 (2.9)	24 (5.9)	ns
*MPL*	6 (1.5)	3 (0.7)	3 (0.7)	ns
*ET Jak2V617F positive*	195 (48.2)	109 (27)	86 (21.2)	0.01
Thrombotic risk score low	132 (32.7)	38 (9.4)	94 (23.3)	< 0.01
Thrombotic risk score high	272 (67.3)	179 (44.3)	93 (23.0)	< 0.01
Comorbidities at diagnosis, *N* (%)	283 (70.0)	157 (38.8)	126 (31.2)	ns
CV-AEs before MPN diagnosis, *N* (%)	268 (66.3)	216 (53.5)	52 (12.8)	< 0.01
*Ischemic heart disease*	31 (7.7)	20 (4.9)	11 (2.7)	ns
*Peripheral arterial disease*	14 (3.5)	7 (1.7)	7 (1.7)	ns
*Cerebrovascular event*	28 (6.9)	19 (4.7)	9 (2.2)	ns
*Atrial fibrillation*	25 (6.2)	15 (3.7)	10 (2.5)	ns
*Deep vein thrombosis*	19 (4.7)	11 (2.7)	8 (2.0)	ns
*Other*	17 (4.2)	9 (2.2)	8 (2.0)	ns
Diabetes mellitus, *N* (%)	51 (12.6)	30 (7.4)	21 (5.2)	ns
Dyslipidemia, *N* (%)	140 (34.6)	77 (19.0)	63 (15.6)	ns
Hypertension therapy, *N* (%)				
RASi		147 (67.7)		
*ARBs*		87 (40)		
*ACEi*		59 (27.1)		
*RASi without association*		116 (53.4)		
Calcium antagonist		52 (23.9)		
Other*		101 (46.6)		
MPNs therapy				
*Low-dose aspirin*	292 (72.3)	148 (36.7)	144 (35.6)	ns
*Phlebotomy*	121 (30.0)	70 (91.0)	51 (0)	ns
*Cytoreduction therapy*	254 (62.9)	159 (80.5)	98 (54.2)	< 0.01
*IFN-2α*	1 (0.2)	1 (0.8)	0 (0.0)	ns
TAEs events after MPNs diagnosis, *N* (%)	61 (15.0)	39 (9.7)	20 (4.9)	0.03
*Ischemic Heart Disease*	14 (3.5)	10 (2.4)	4 (0.9)	ns
*Peripheral arterial disease*	12 (2.9)	6 (1.5)	6 (1.5)	ns
*Cerebrovascular event*	15 (3.7)	12 (2.9)	3 (0.7)	ns
*Deep Vein Thrombosis*	18 (4.4)	11 (2.7)	7 (1.7)	ns

Abbreviations: *MPNs* Chronic myeloproliferative neoplasms; *CV-AEs* Cardiovascular adverse events; *RASi* Renin-angiotensin inhibitors; *ARBs* Angiotensin receptors blockers; *ACEi* Angiotensin-converting enzyme inhibitors; *TAEs* Thrombotic-adverse events; *ET* Essential thrombocythemia

^*^Included thiazide diuretics, beta-blockers and doxazosin

Among 217 patients with hypertension, 147 of them (67.7%) were treated with RAS inhibitors. They were administered alone in 116 cases (53.4%) or associated with other anti-hypertensive agents only in 31 cases (14.2%). Fifty-nine patients (27.1%) received ACEi, and eighty-seven (40%) patients were treated with ARBs. Fifty-two patients (23.9%) received calcium channel blockers, and in 101 (46.6%) patients, other agents such as thiazide diuretics, beta-blockers, and doxazosin were utilized. A total of 254 (62.9%) patients received cytoreductive therapy; most were treated with hydroxycarbamide. In the PV cohort, phlebotomy was administered in 121 (91%) patients. Out of the 292 patients (72.3%) received a low dose of aspirin. Among these, aspirin was started at the time of MPN diagnosis in 123 cases.

In the MPN cohort, 61 (15%) TAEs were collected after a median of 6.5 years (range 1–14) after the MPN diagnosis. The 15-year cumulative incidence of TAEs events was 52.1 ± 6.8%. TAEs events were represented by 14 (3.5%) ischemic heart disease, 12 (2.9%) peripheral arterial disease, 15 (3.7%) cerebrovascular events, and 18 (4.4%) deep vein thrombosis.

**Fig. 1:**
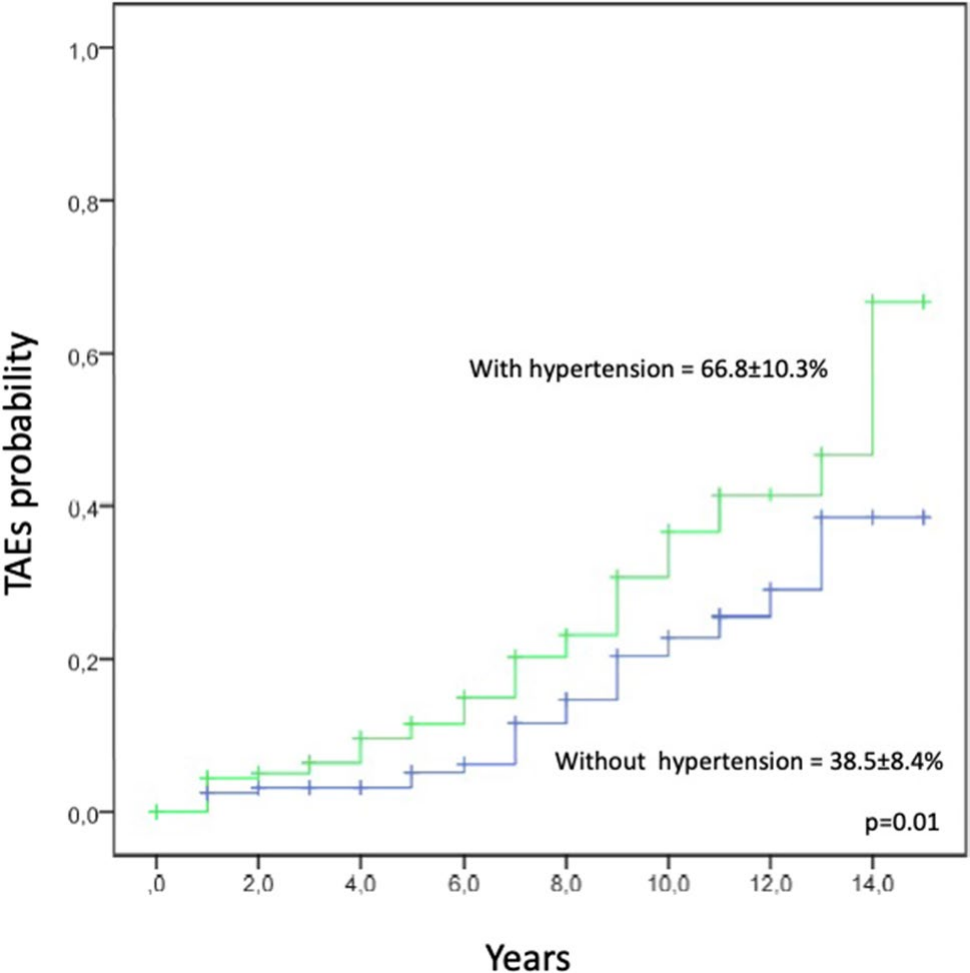
Cumulative incidence of thrombotic adverse events in patients with Polycythemia Vera and Essential Thrombocythemia, according to the hypertensive status

The 15-year cumulative incidence of TAEs was significantly higher in patients with hypertension (66.8 ± 10.3% vs 38.5 ± 8.4%; HR = 1.83; 95%CI 1.08–3.1) Fig. 1. Multivariate analysis showed that PV diagnosis and hypertension were independently associated with a higher risk of developing TAEs (HR = 3.5; 95%CI 1.928–6.451, *p* < 0.001 and HR = 1.8; 95%CI 0.983–3.550, *p* = 0.05, respectively). In multivariate analysis, the diagnosis of PV confirmed a significant predictive role in developing TAEs (HR = 4.4; 95%CI 1.92–10.09, *p* < 0.01), also considering only MPN patients with hypertension. We also found that the use of RASi showed a protective effect from TAEs both in the whole cohort of MPN with hypertension (HR = 0.46; 95%CI 0.21–0.98, *p* = 0.04) and in the subgroup of thrombotic high-risk score patients (HR = 0.49; 95%CI 0.24–1.01, *p* = 0.04). In particular, patients with ET and a high risk of thrombosis seem to benefit most from RASi treatment (HR = 0.27; 95%CI 0.07–1.01, *p* = 0.03). No association was found between TAEs and the duration of hypertension.

## Discussion

The main goal of managing MPNs is to prevent thrombotic incidents. According to the conclusions of the European Collaboration on Low-dose Aspirin (ECLAP) study, cardiovascular mortality is the primary cause of death in PV [22]. Furthermore, the incidence of thrombosis in PV ranges from 10 to 40%, whereas in ET, it ranges from 10 to 25% [2, 23]. As per the Swedish Cancer Register's data, a comprehensive study was conducted on more than 11,155 individuals diagnosed with MPNs and 44,620 healthy controls. The results indicated that MPN patients had a significantly higher risk (4.9 fold) of arterial thrombosis than the healthy controls [24]. The JAK2V617F mutation has been linked to a heightened susceptibility to thrombotic risk. This mutation is present in 90% to 95% of patients with PV, which may partially explain why they have a higher incidence of thrombotic events. [15]. In addition, recent research has shown that platelet activation significantly impacts the pathogenesis of thrombosis in MPN [25]. To prevent TAEs, a precise approach is necessary to target specific blood levels of PLT and HCT. This includes lowering HCT levels and managing thrombocytosis through cytoreductive therapy and anti-platelet treatments [7]. Administering aspirin has proven effective in reducing the risk of thrombosis in patients with PV/ET [26].

The development of TAEs is also promoted by modifiable CV risk factors such as dyslipidemia, hypertension, diabetes, and smoking. This is common in the adult population, particularly among those in the age group where MPN patients are often diagnosed [27]. Multiple studies indicate that individuals diagnosed with ET and having one or more CV risk factors are more susceptible to experiencing thrombotic events [15, 17, 28, 29]. Among these, hypertension is a prevalent and pressing medical condition that affects individuals globally. Elevated blood pressure levels can result in detrimental cardiovascular implications, amplifying the risk of mortality [30, 31]. Despite this, a limited number of studies have been conducted to investigate the specific effects of hypertension on MPN. Lekovic et al. found a correlation between arterial thrombotic events in individuals with ET and hypertension [17]. An elegant study of 816 patients with PV showed that 41.9% had a Charlson comorbidity index (CCI) of 1 or higher.

Furthermore, 45.5% of patients had a BMI of 25 or more at the time of PV diagnosis. The authors found that high CCI scores, a BMI above 25, and hypertension were linked to a higher risk of thrombosis [20]. Although managing modifiable cardiovascular risk factors has been included in redefined scores, it only plays a minor role in treating MPNs [28].

The renin-angiotensin-system (RAS) plays a significant role in regulating blood pressure and can contribute to the onset of hypertension and atherosclerosis [32]. This system relies on the enzymatic action of renin to carry out its vital functions. Specifically, renin catalyzes the cleavage of angiotensinogen, a plasma protein, into angiotensin I. This inactive peptide is then transformed into angiotensin II, a potent vasoconstrictor critical in regulating blood pressure and fluid balance. Overall, the enzymatic activity of renin is essential for maintaining proper physiological function and homeostasis. The process usually occurs in the lungs’ endothelial cells and is performed by the non-specific protease ACE1 [33]. RAS may contribute to the development of atherosclerosis by affecting vascular inflammation and hemostasis through oxidative stress. This can lead to endothelial dysfunction and an increased risk of thrombin generation, which may further exacerbate the progression of atherosclerosis [34]. On the other hand, blocking the RAS components has been shown to lower the risk of developing atherosclerosis and dying from cardiovascular disease in human studies. Therefore, it has been suggested that RAS inhibition could offer benefits against atherosclerosis beyond just lowering blood pressure [35].

Previous research has indicated the existence of intrinsic RAS within the bone marrow (BM), which plays a crucial role in managing the process of hematopoiesis [36]. Furthermore, it has been observed that in clonal hematopoiesis of PV and ET, the angiotensinogen gene is upregulated and significantly expressed in JAK2V617F-positive patients [37]. After analyzing the data, it suggests the link between MPNs, hypertension, and occlusive events. Furthermore, this connection could have significant implications for treatment. A recent analysis of patients who participated in the ECLAP study suggests that interfering with the RAS system is connected to decreased hematocrit levels, despite no observed effects on reducing TAE events [38].

We analyzed the role of hypertension in a large cohort of 404 MPN patients and found that the 15-year cumulative incidence of TAEs was significantly higher in patients with hypertension (66.8 ± 10.3% vs 38.5 ± 8.4%; HR = 1.83; 95%CI 1.08–3.1). The association between hypertension at MPN diagnosis and TAEs was confirmed in multivariate analysis, together with the diagnosis of PV, as a strong predisposing CV risk factor. This finding suggests careful attention to MPN patients with hypertension. Ideally, these patients require the availability of a cardio-oncology facility, being cardio-oncology a discipline based on the collaboration between cardiologists, hematologists, and other medical specialists to prevent, monitor, diagnose and treat TAEs in MPN patients.

Moreover, we found a protective association between the use of RAS inhibitors and the reduction in TAEs in our cohort of MPN patients. In particular, patients with ET and thrombotic high-risk seem to benefit most from RASi treatment (HR = 0.27; 95%CI 0.07–1.01, *p* = 0.03). It may be worth considering certain classes of hypertensive drugs as electives for patients with MPN, such as ACEi and ARBs. Another interesting aspect is the potential use of new drugs in treating PV, such as the hepcidin mimetic [39]. Hepcidin is a peptide hormone primarily responsible for regulating the balance of iron in the body and also impacts erythropoiesis [40]. Recent studies have revealed that hepcidin inhibits the enzyme renin, suggesting a potential role in controlling blood pressure [41]. These findings suggest possible implications for further research and clinical considerations in hematopoietic disorders.

In conclusion, to improve the treatment of patients with MPN, it is crucial to pay close attention to their cardiovascular risk factors, as these factors play a significant role in the complications of the disease. A more targeted approach could provide more effective and personalized care for patients with MPN. Although the study's retrospective nature poses limitations, the robust connections between the RAS system and hematological disorders make it crucial to conduct a more comprehensive analysis of the effects of RAS inhibitors on MPN.

## Data Availability

Medical charts and database are available at Hematology Unit, Businco Hospital, ARNAS Brotzu Cagliari, Cagliari, Italy.
